# Multivariate Approach for Studying Interactions between Environmental Variables and Microbial Communities

**DOI:** 10.1371/journal.pone.0050267

**Published:** 2012-11-26

**Authors:** Xinhui Wang, Marinus J. C. Eijkemans, Jacco Wallinga, Giske Biesbroek, Krzysztof Trzciński, Elisabeth A. M. Sanders, Debby Bogaert

**Affiliations:** 1 Department of Pediatric Immunology and Infectious Diseases, UMC Utrecht, Utrecht, The Netherlands; 2 Julius Center for Health Sciences and Primary Care, UMC Utrecht, Utrecht, The Netherlands; 3 Centre for Infectious Disease Control, National Institute for Public Health and the Environment, Bilthoven, The Netherlands; University of Iowa Carver College of Medicine, United States of America

## Abstract

To understand the role of human microbiota in health and disease, we need to study effects of environmental and other epidemiological variables on the composition of microbial communities. The composition of a microbial community may depend on multiple factors simultaneously. Therefore we need multivariate methods for detecting, analyzing and visualizing the interactions between environmental variables and microbial communities. We provide two different approaches for multivariate analysis of these complex combined datasets: (i) We select variables that correlate with overall microbiota composition and microbiota members that correlate with the metadata using canonical correlation analysis, determine independency of the observed correlations in a multivariate regression analysis, and visualize the effect size and direction of the observed correlations using heatmaps; (ii) We select variables and microbiota members using univariate or bivariate regression analysis, followed by multivariate regression analysis, and visualize the effect size and direction of the observed correlations using heatmaps. We illustrate the results of both approaches using a dataset containing respiratory microbiota composition and accompanying metadata. The two different approaches provide slightly different results; with approach (i) using canonical correlation analysis to select determinants and microbiota members detecting fewer and stronger correlations only and approach (ii) using univariate or bivariate analyses to select determinants and microbiota members detecting a similar but broader pattern of correlations. The proposed approaches both detect and visualize independent correlations between multiple environmental variables and members of the microbial community. Depending on the size of the datasets and the hypothesis tested one can select the method of preference.

## Introduction

Microbial communities naturally populate our body surface, though differ with respect to composition and function between body sites. Human microbiota consist of approximately 100 trillion bacterial cells that outnumber host cells with a factor ten or more [1], [2]. Microbiota have important functions in health, for example by influencing nutritional processes [3], [4], or affecting susceptibility to inflammatory processes like inflammatory bowel diseases [5], [6], [7], [8], [9], pneumonia [5], [6], [7], [8], [9], and asthma [5]. Nevertheless, the determinants of human microbial communities are still poorly understood.

The rapid development of deep sequencing technologies has enabled us to measure in detail the composition of even most complex microbial communities in various environments, which has lead to a new world of knowledge [10], [11]. An increasing number of articles are being published on the human microbiome, showing correlations between microbiota profiles and different environmental or health characteristics. For example, Gill *et al.* described that the distal gut microbiome is significantly enriched for several metabolic functions [12]. Turnbaugh *et al*. observed that the composition of the gut microbiome differed in twins when comparing obese and lean individuals [13]. Jakobsson *et al*. identified changes in microbial communities of the oropharynx and faeces after antibiotic treatment [14]. Bogaert *et al*. recently reported the presence of seasonal changes in the nasopharyngeal microbiota of young Dutch children [15]. All these studies showed that variability in species dominating these microbial communities may be highly related to environmental factors and the person’s health status. Therefore, thorough investigation of interactions between environmental variables, human health status and microbiota composition will most likely contribute to better insight into health and disease.

One of the factors that limit progress in understanding the human microbiome dynamics is the lack of suitable statistical and visualization tools to assess determinants of these ecologically communities. Currently, most of the studies focus on the analysis of only one or two potential variables of community profiles. However, for a more complete understanding of interactions and their effect sizes, multiple environmental and epidemiological variables need to be taken into account to correct for simultaneous independent and synergistic effects. For example, in respiratory microbiota a strong driver of community composition seems season. It is therefore very difficult to detect independent effects of additional parameters that follow a seasonal pattern,such as respiratory viruses, on microbiota composition. To this purpose, multivariate analysis is required [15].

Several general multivariate techniques have previously been used to identify interactions between environmental variables and microbial communities: Ye *et al*. [16] and Sinkko *et al*. [17] used canonical correlation analysis (CCA) to detect the relationships between bacterial community composition and chemical parameters. CCA determines linear combinations of the variables and community members which have a maximum correlation, however it is difficult to interpret the individual correlation between each environmental variable and community member as well as their effect sizes. Moreover, weak correlations are difficult to identify. There is ongoing discussion about proper visualization because of the inherently complicated results of CCA [18], hence limiting the application of this technique [19]. Furthermore, the descriptive nature of these approaches does not allow for specific hypotheses testing.

In this study, we propose to study epidemiological background variables (metadata) in relation to microbial communities using two approaches that only differ in their initial step:

- we use CCA to make a selection of environmental variables and microbial community members in the microbiota, followed by a classical multivariate regression analysis to determine the size and direction of the individual correlations, and visualize the effect size and direction of those correlations in a heatmap;
- we use univariate or bivariate regression analysis to make a selection of environmental variables and microbial community members, followed by a classical multivariate regression analysis to determine the size and direction of the individual correlations, and again visualize the effect size and direction of those correlations in a heatmap.

We illustrate the use of these methods by applying them to a complex dataset containing epidemiological variables and accompanying microbiota profiles of 96 nasopharyngeal samples of children processed by 16S-rDNA-based sequencing [15].

**Figure 1:**
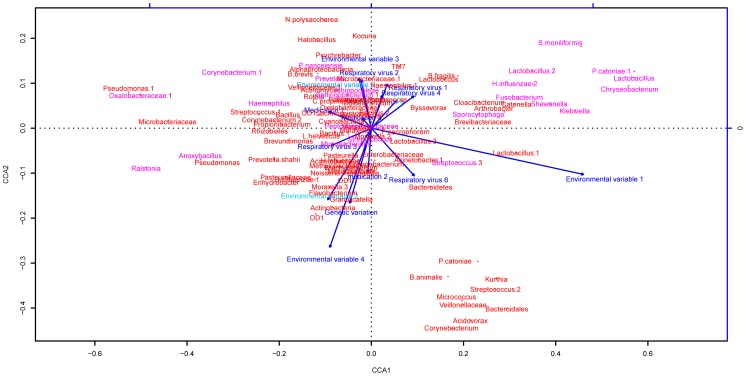
Correlations between variables and microbiome composition at OTU level identified by the different types of analysis. In figure 1, results from Canonical Correlation Analysis (CCA) are depicted, where each variable is plotted with the weight of this variable from the first order and second order variates as coordinates. Pairs of variables with relatively large weights in the same direction represent positive correlations and variables whose weights have opposite directions exhibit inverse correlations.After applying the canonical correlation analysis with a cut off p-value <0.15, 12 external variables (in blue color) and 31 OTUs (in old rose color) remained as potential determinants of microbiota composition.

## Methods

### Data Set

#### Microbiota data

We used a dataset of metagenomic data as well as metadata of 96 children 18 months of age and participating in a randomized controlled trail as described previously [15]. Nasopharyngeal samples were obtained and processed by 16S-rDNA-based 454-sequencing as in [15]. In short, differences between each unique sequence and sequences in the SILVA database [20] were calculated using the GAST algorithm to obtain taxonomic information [21]. Subsequently, operational taxonomic units (OTUs) were created by aligning the approximately 1.2 million unique sequences, using DOTUR [22] to create clusters at a 3% level. Because a bar-coded approach was used, bacterial diversity and composition could be determined for each individual sample. In total 13 taxonomic phyla and approximately 250 OTUs were found. Data on phyla level as well as the 100 most predominant OTUs were subsequently further studied by multivariate analyses. To be able to compare the relative abundance of OTUs among samples, the data were normalized for sequence reads per sample (about 10.000 reads per sample). Because the normalized data contained relative frequencies, resulting in a value between 0 and 1, and many OTUs are low abundantly present, we expected the data to have a right-skewed distribution. Therefore, in all statistical analyzes, logarithmically transformed (log10) data were used.

**Figure 2:**
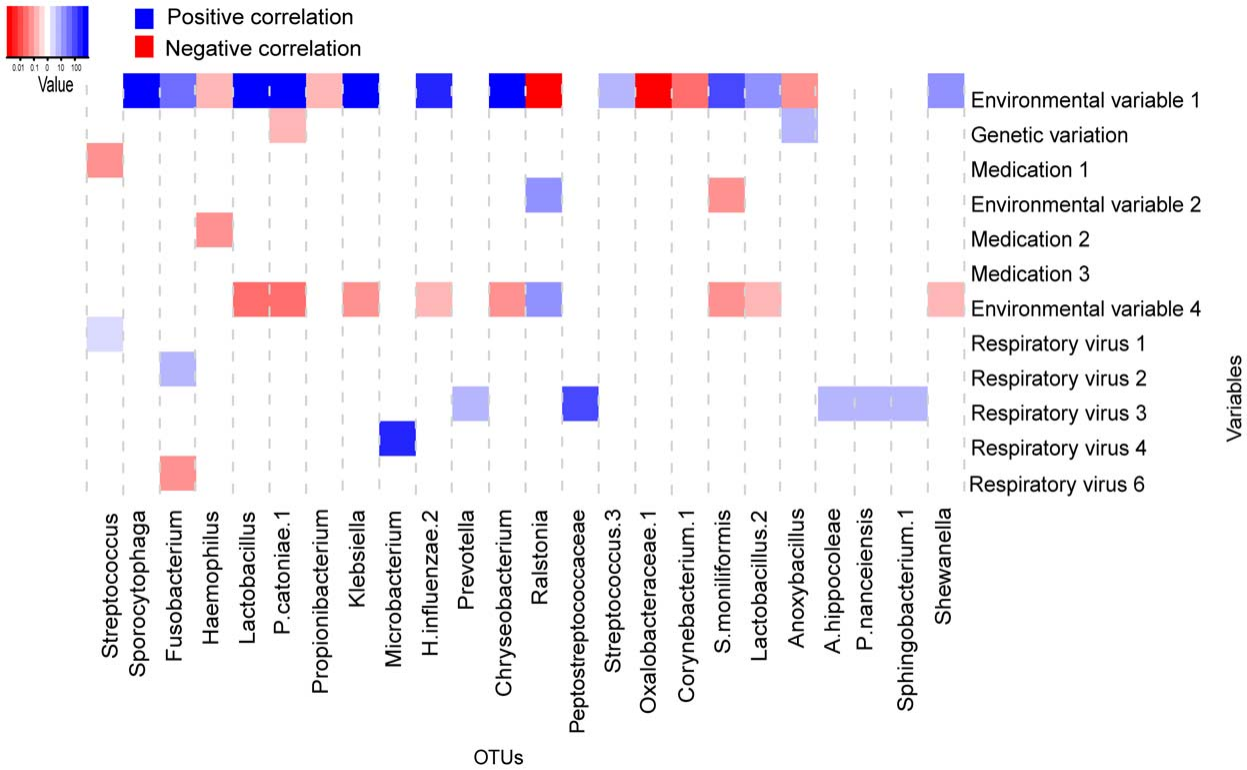
Represent the results from multivariate logistic regression analysis following CCA. Significant correlations are depicted between the by CCA selected 12 variables and 31 OTU’s. The x-axis depicts the respective OTUs and the y–axis the 12 variables tested. The heatmap shows significant correlations (p-value less than 0.05) from multivariate analysis. The colour represents the effect size and direction of the correlation. Blue squares show positive changes in relative abundance, whereas red squares show negative correlations. The intensity of color correlates with the magnitude of the (log) fold change value (see colour key).

#### Metadata

In addition to the nasopharyngeal samples, a questionnaire was obtained containing information regarding risk factors for colonization and infection with respiratory viruses and bacteria. From this questionnaire, 15 potential determinants of community profiles were chosen that had previously been shown to be determinants of colonization with certain bacterial species (see reference [8], [9] for a description of the accompanying metadata), including five environmental variables (daycare, feeding type, season, sibling and smoke exposure), three medical variables (recent antibiotic use, use of bronchodilating medicine and PCV-7 vaccination), one genetic variable (sex) and six variables representing presence/absence of common respiratory viruses (adenovirus, bocavirus, KI and WU virus, para-influenza virus and respiratory syncytial virus). All variables are quantitative data represented by 0 (absent) or 1 (present).

**Figure 3:**
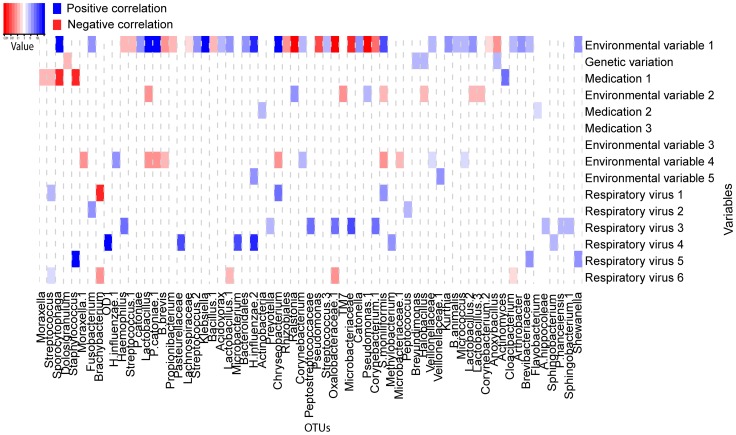
Represents the results from univariate regression analysis. Significant correlations between 15 variables and the top 100 OTUs are depicted. The x-axis depicts the respective OTUs and the y–axis the 15 variables tested. The heatmap shows significant correlations (p-value less than 0.05) from univariate analysis. Blue squares show positive changes in relative abundance, whereas red squares show negative correlations. The intensity of colour correlates with the magnitude of the (log) fold change value (see colour key).

**Figure 4:**
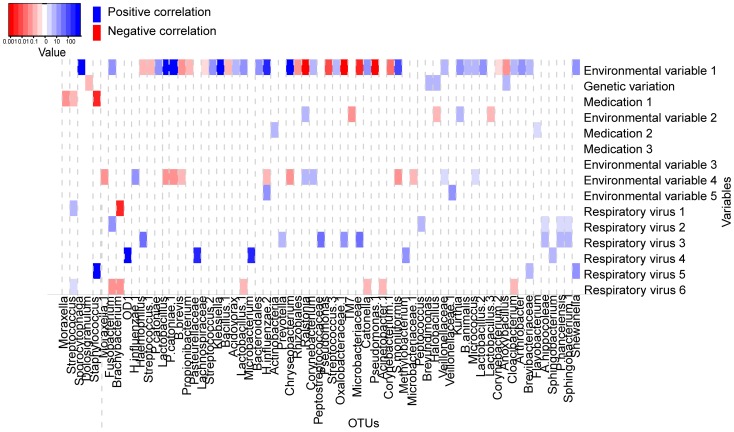
Represents the results from bivariate regression analysis. Significant correlations between 14 variables and the top 100 OTUs are depicted after correcting for the correlation between season and OUT composition. The x-axis depicts the respective OTUs and the y–axis the 15 variables tested. The heatmap shows significant correlations (p-value less than 0.05) from univariate analysis. Blue squares show positive changes in relative abundance, whereas red squares show negative correlations. The intensity of colour correlates with the magnitude of the (log) fold change value (see colour key).

### Exploratory Methods

#### Canonical correlation analysis

For the first approach, the data was explored with canonical correlation analysis (CCA). CCA seeks linear combinations of the selected variables correlating with linear combinations of the 100 OTUs. The main idea of this method is to find strong correlations between these two datasets. We used the Canonical correlation analysis to test for possible correlations between individual external variables and the overall microbial community, plus correlations between all external variables and each individual OTU. We performed CCA using the CCorA function in the vegan package (software R version 2.7) to detect the interactions between the selected metadata and the given microbiota dataset at OTU level (100 OTUs) and used the envfit function to get the p-value of correlation of each variable with overall bacterial communities and the p-value of each correlation between each OTU and all variables. The first order and second order weights of the variables and phyla of such combinations were plotted. The canonical correlation analysis was used to eliminate variables and microbial community members without any correlations with one another. These variables and OTUs were excluded from the next step of our multivariate approach. For this dataset the cut-off for significance was set at p<0.15, which for a single parameter is equivalent to the commonly used Akaike’s Information Criterion (AIC) for model selection.

**Figure 5:**
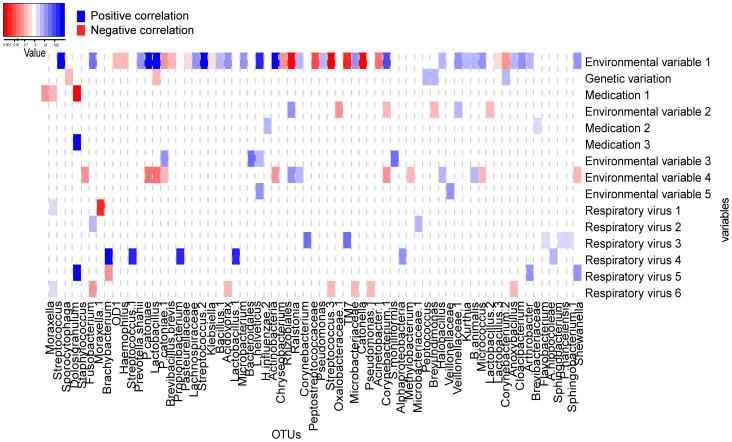
Represents results from multivariate regression analysis following bivariate regression analysis. The x-axis depicts the by bivariate regression selected 68 OTUs and the y–axis the selected variables. The heatmap shows significant correlations (p-value less than 0.05) from multivariate analysis. Blue squares show positive changes in relative abundance, whereas red squares show negative correlations. The intensity of colour correlates with the magnitude of the (log) fold change value (see colour key).

#### Univariate analysis

In the univariate analysis, the effect of each variable on the relative presence of each individual OTU was tested using analysis of variance (function lm and ANOVA in software R 2.7). For each OTU, only the variables with a p-value less than 0.15 in univariate analysis were selected for consecutive multivariate analysis. For some data sets, if there is a known predominant determinant (in our data set this is season, but for example also age might be a major determinant), bivariate analysis can be performed to detect more subtle correlations independent from the predominant one that may otherwise have stayed unnoticed. In bivariate analysis, the effect of each variable is tested against the OTU dataset while corrected for the predominant determinant or confounder of the microbiota (such as age or season). Only variables correlating with microbiota members (for our dataset defined as all variables correlating with a given OTU with a p-value of less than 0.15) were selected for consecutive multivariate analysis.

#### Multivariate analysis

In multivariate analysis, the selected variables were subjected to a multiple linear model [23] (function lm and ANOVA in software R version 2.7). For our relatively small dataset that we use for illustrating purposes only, the statistical significance was set at a p-value less than 0.05 to include the most pronounced trends in the multivariate analysis. For analysis of larger datasets we propose to use a correction for multiple testing, for example through False Discovery Rate (FDR) [24], [25] reducing the risk of false positive results.

#### Visualization

We calculated the direction and effect-size of all observed correlations between variables and OTUs based on the regression coefficients of the fitted linear model. Note that for calculation of the effect-size back-transformation of OTU data is needed since the dataset contained log-transformed data for statistical purposes because of right-skewed distribution (see method section: microbiota data) The fold change is always a positive value, where fold changes above 1 represent increases in relative abundance of a given OTU with a given determinant, whereas fold changes between 0 and 1 represent decreases in OUT abundance with a given determinant.

Since a multivariate model is made for each OTU that correlates with one or more of the environmental parameters, a multitude of models need to be interpreted. To aid interpretation of these results, significant p-values or corresponding fold changes of all models together will be presented in a single heatmap (function heatmap.2 from package gplots in software R 2.7),.

## Results

### 

#### Canonical correlation analysis

We used the canonical correlation analysis to detect the correlations between the 15 selected environmental variables and the 100 most common microbial community members (OTUs). The results are presented in a heatmap format in figure 1. After applying the canonical correlation analysis with a cut off p-value <0.15, 12 external variables and 31 OTUs remained as potential determinants of microbiota composition.

#### Method (i) Multivariate regression after canonical correlation analysis

Multivariate regression was performed on the by canonical correlation identified determinants and OTUs. All significant correlations between the selected individual variables (12 variables) and bacteria at OTU level (31 OTUs selected with canonical correlation analysis) are shown using a heatmap in Figure 2. In total, 43 significant correlations between individual variables and OTUs were observed in multivariate analysis; 23 positive and 20 negative correlations. The fold change of the significant correlations at OTU level is depicted in the figure. The red squares represent negative correlations with a fold change ranging from 0.001 to 0.228 whereas blue represents positive correlations with a fold change ranging from 1.363 to 1195, respectively.

#### Univariate and bivariate analysis

For our dataset, we performed univariate analysis to select for combinations of variables and OTUs to enter in the multivariate model. Following, we performed bivariate analysis testing for potential correlations between each determinant and each OTU again, independent of the effect of environmental variable 1 (i.e. season). Figure 3 represents the significant correlations as identified by univariate analysis. Figure 4 represents the significant correlations as identified by bivariate analysis (p<0.05). We observed 63 positive correlations and 36 negative correlations by univariate analysis and 62 and 36 correlations by bivariate analysis The effect size and direction of the correlation is presented by the fold change value and color.

#### Method (ii) Multivariate regression after univariate or bivariate analysis

Figure 5. represent the significant correlations (p-value less than 0.05) identified by multivariate analysis following bivariate analysis. In total, 106 correlations were identified. We found 82 significant interactions between variables and OTUs in both univariate and multivariate analysis, whereas 16 correlations were found by univariate, and 24 correlations were found by multivariate analysis only.

## Discussion

In this paper, we propose two multi-step multivariate approaches for studying the potential correlations between multiple external variables and complex microbial community profiles. We used both approaches on a dataset containing microbial community profiles of nasopharyngeal samples of 96 children that were 18-months of age and accompanying metadata. We showed that the multivariate approaches could detect the effect of presence of respiratory viruses on microbiota composition against the background of a large seasonal driving force. These approaches allow for easy interpretation of correlations, and can be applied by a broad range of biomedical researchers who might not all have access to specialized bioinformatics support.

CCA can be used for integrated analysis of multiple complex datasets like microbiota data of large number of samples and accompanying extensive metadata, and is therefore a powerful tool. However, the results of canonical correlation analysis (CCA) are generally difficult to interpret. No specific hypothesis testing on combinations of individual determinants and OTUs could be applied, and the statistical strength of the correlations is difficult to evaluate. However, when combined with classical multivariate regression analysis in approach (i), visual interpretation of individual independent variable-OTU correlations and their direction and effect size was achieved.

The other proposed approach (ii), i.e. univariate, bivariate analysis followed by multivariate regression analysis, detected similar correlations as approach (i). For example both approaches were able to confirm the positive correlation between the presence of specific respiratory viruses (virus 1 and virus 6) and density of Streptococcus as was previously observed by us and others using conventional cultures ([26]). The fact that slightly different approaches yield similar results underlines the robustness of the findings from both approaches. However, approach (ii) detected some additional correlations, suggesting a broader picture or less strict analysis.

Comparing results from approach (i) and (ii) with the results of univariate analysis clearly illustrates the drawback of univariate analysis: it does not correct for simultaneous or dependent and synergistic effects. For instance, univariate analysis only did not detect a correlation between virus 6 and Bacteroidetes, whereas this strong correlation was identified with both approach (i) and (ii). Univariate analysis detected a correlation between virus 1 and the OTUs *Streptobacillus moniliformis* and *Chrysobacterium*, whereas these correlations were not identified in multivariate analyses of both approach (i) and (ii). The same correlations were however observed between those bacteria and season in univariate analysis, which persisted after multivariate analysis, suggesting this viral-bacterial correlation depended on a seasonal distribution rather than a direct relationship.

As we stated in the results section, if there is a known predominant determinant, then bivariate analysis following univariate will be helpful to make a more precise selection to enter into multivariate regression analysis, since it allows for identification of less strong correlations to enter in MVA that otherwise would have been ignored. This is illustrated by Figures 3 and 4, where results from uni- and bivariate analysis are depicted: it is clearly shown, especially for the respiratory viruses, that after correction for season, additional correlations appear. This is probably caused by the fact that respiratory viruses follow a seasonal pattern, and therefore might be wrongly missed when univariate correlations are selected only, depending on the sign and size of the correlation between season and OTU abundance. When no known predominant determinant is expected or identified for a given dataset, the univariate analysis as a selection tool for multivariate analysis will suffice.

We have used the results from the multivariate analysis to calculate the size and direction of the identified correlations using fold change. Most significant correlations identified in the multivariate analysis had large effect sizes (fold change greater than 2 or less than 0.5). The fold change provides the direction and size of the effect of a determinant and is, therefore, extremely valuable for clinical or practical interpretation of the results. Using heatmaps, one can easily interpret the achieved results.

We note that the proposed multivariate analysis can be applied to any data set to identify (multiple) independent determinants or parameters of bacterial communities. The bacterial communities can be obtained from other sites within the human body (e.g., skin, gut, or oral cavity) or environmental settings (e.g., soil, deep sea and atmosphere). The designed multivariate analysis approach can also be used on qualitative data (e.g., the presence or the absence of OTUs, using logistic regression analysis rather than regression analysis in MVA). In a qualitative analysis some information will be lost (i.e., the absolute number of abundance), and in a quantitative analysis the low abundant reads will create erroneous ratios due to higher standard deviations. A natural extension of the proposed method would be to use a quantitative analysis (ratio) for the common OTUs and qualitative analysis for the low prevalent OTUs. Although the proposed approach performed well on our dataset, there is a risk of introducing errors when non-linear correlations are present, as the proposed multivariate approach considers all variables in a linear correlation. Another natural extension of the proposed method in the future would be the inclusion of detection of non-linear correlations.

In summary, this paper describes two multivariate approaches, which can be used to detect multiple independent correlations between environmental variables and microbial communities and correct for potential confounders. Approach (i), using CCA to select the variables for multivariate regression, resulted in a more specific range of interactions when compared to selection by uni- or bivariate analysis, suggesting a more strict selection of parameters. For both approaches, the fold change is calculated to show direction and effect size of the identified correlations. Heatmaps are used to visualize the significance and effect size of the multivariate findings incorporating all interactions between external parameters and the overall microbial community. This will allow for more complex analyses reducing bias by including concomitant effects of multiple determinants.
